# The shortcomings of accurate rate estimations in cultivation processes and a solution for precise and robust process modeling

**DOI:** 10.1007/s00449-019-02214-6

**Published:** 2019-09-20

**Authors:** B. Bayer, B. Sissolak, M. Duerkop, M. von Stosch, G. Striedner

**Affiliations:** 1 Department of Biotechnology, University of Natural Resources and Life Sciences, Vienna, Austria; 2 Bilfinger Industrietechnik Salzburg GmbH, Salzburg, Austria; 3 School of Chemical Engineering and Advanced Materials, Newcastle University, Newcastle upon Tyne, UK

**Keywords:** Bioprocess development, Cubic smoothing spline, Fed-batch fermentation, Growth rate, Substrate uptake rate

## Abstract

The accurate estimation of cell growth or the substrate consumption rate is crucial for the understanding of the current state of a bioprocess. Rates unveil the actual cell status, making them valuable for quality-by-design concepts. However, in bioprocesses, the real rates are commonly not accessible due to analytical errors. We simulated *Escherichia coli* fed-batch fermentations, sampled at four different intervals and added five levels of noise to mimic analytical inaccuracy. We computed stepwise integral estimations with and without using moving average estimations, and smoothing spline interpolations to compare the accuracy and precision of each method to calculate the rates. We demonstrate that stepwise integration results in low accuracy and precision, especially at higher sampling frequencies. Contrary, a simple smoothing spline function displayed both the highest accuracy and precision regardless of the chosen sampling interval. Based on this, we tested three different options for substrate uptake rate estimations.

**Electronic supplementary material:**

The online version of this article (10.1007/s00449-019-02214-6) contains supplementary material, which is available to authorized users.

## Introduction

State variables, such as biomass, substrates, and product, are quantified via off-line measurements during cultivation processes of microbial, mammalian and yeast cells to understand how the process states evolve. To shed light into the biological subsystem, i.e., the cell state, as well as the metabolism [4, 6, 8, 12] or to compare different cultivations on the biological level, e.g., for media selection or cell line development [13, 16, 19], specific production/consumption rates are a necessity.

### Principle approaches to rate estimation

There are several approaches for estimating rates of a bioprocess [7, 15, 21]. A very simple method is to calculate the first derivative of a cubic smoothing spline function [15, 21]. The result is a continuous rate over the whole course of a bioprocess such as a fed-batch process, where for every time point, a rate value can be derived.

Although the applicability of this non-parametric method on bioprocess data is known for a longer time [3, 15], it still does not seem to be the method of choice for researchers in upstream bioprocess engineering, or related fields of biology. In most cases, the integral approach, a simple stepwise integral estimation is used [5, 10, 11, 25]. Hereby two measurements, one derived from sampling time point t_i_ and the other from sampling time point *t*_*i*+1_, are considered to estimate a rate for this interval (*t*_*i*_, *t*_*i*+1_). The same methodology is then applied to the next interval (*t*_*i*+1_, *t*_*i*+2_) and so on, estimating one rate value for each time interval, resulting in a trend over the course of the cultivation process. This, in turn, means that the rate is assumed to be constant for each sampling interval, for which it was calculated, independent on its length.

### Parameters impacting rate estimation quality

Some parameters do have a high impact on the outcome of these rate estimations and if treated in the wrong way result in false estimations. For instance, dynamic process trends can remain unnoticed, e.g., if the sampling frequency is too low. In addition, if larger measurement errors are present, the rate is not feasible to describe the process anymore due to this inaccuracy. This can lead to a reduction of the accuracy of the rates and to a reasonably weakened hypothesis on the influences of certain variables or parameters. To make the calculations more applicable, different smoothing approaches for rates can be used. An often described and simple method is the moving average [9, 26]. Here, the rates from several sampling points are smoothed by taking the average value from a sampling window. In addition, more advanced moving average filters such as low-pass and Savitzky–Golay were already retrospectively used for rate modeling of bioprocesses [14, 17]. Such advanced filters require settings and appropriate knowledge for the ideal window size and smoothness, which are dependent on the process they are applied on. Using these methods, the true covariance matrix is often underestimated and the lack of automatic constraints for state variables may lead to suboptimal performances [23].

### Accurate estimation of a rate

Key figures existing in every cultivation process are the growth rate µ, which is defined as the time derivative of the logarithm of the change in population size and specific substrate uptake rates, which are feed dependent. Although stepwise integral estimation gives a simple estimation of the growth rates, this calculation possesses several drawbacks. One discrete estimation from one sampling time point to the next one is suboptimal for non-linear trends. Due to inaccurate biomass measurements, which is, in particular, true for cell culture cultivations, cell growth rates vary strongly between the samplings, indicating a false process status. On the other hand, variations in the amount of fed substrate can have substantial impacts on the specific uptake rate estimation due to error propagation. A switch in the cell’s behavior is more likely to happen continuously and not spontaneously. It can be expected that calculations and model building attempts with these obtained biased values can lead to unreliable results containing much noise. To yield better descriptions of cultivation processes continuous rates should be preferred over sudden changes to yield.

Since the “true” rate is not accessible in a real fermentation process, because of the existence of analytical measurement errors [20] and biological differences from cultivation to cultivation, we present a simulated case study, at which linear and inhibited cell growth were simulated in-silico. Noise was added to the dataset to mimic a range of typical analytical measurement errors. 100 single fed-batch processes were simulated to obtain a statistical meaningful dataset. We compared the performance of the stepwise integral estimation including post-smoothing with a simple moving average with the cubic smoothing spline function. Hereby, different sampling intervals and analytical measurement errors have been simulated and both approaches were elucidated with respect to their precision and accuracy to obtain the real rates. Additionally, we also highlight an optimal solution to describe the substrate uptake rates, since for estimating substrate uptake rates, the feeding rate and feeding substrate concentration need to be taken into account. Any analytical error in this part can have a huge impact on the level of noise in the data.

The unique combination of different rate calculations applied on data with varying sampling frequencies and analytical deviations is very valuable for process understanding and modeling.

## Materials and methods

The detailed cultivation settings for the different simulated in-silico fed-batch fermentations (table 1) and all the necessary equations (Eqs. 1–4) are given in the *Bioprocess Simulation* section of the Online Resource 1.

### Noise generation

To account for process and analytic related variance, randomly generated multivariate normal distributed numbers were added, accounting for different precision levels in each process variable. Such noise was added to volume (1%), substrate (1%), and biomass, for every sampling point. For the biomass, five different levels of coefficient of variation (CV) were utilized (2.5, 5, 7.5, 10 and 12.5%). The CV (Eq. 1) is the standardized standard deviation, independent of the extent of the value and, therefore, a good estimation for accuracy:

1 $${\text{CV }} = \frac{{\upsigma }}{{\bar{X}}} \times 100.$$

The CV describes the magnitude of variation for 68.2% of the data with the standard deviation σ and the average value $\bar{X}$.

### Stepwise integral estimation

The most commonly used method, the stepwise integral estimation, of calculating specific growth rates using the measured cell dry mass is described in the following equation:

2 $$\mu = \frac{{\ln \left( {\frac{X\left( t \right)}{{X\left( {t - 1} \right)}}} \right)}}{{{\text{d}}t}}.$$

As in Takuma et al. [22], *µ* is estimated for each time interval between two measurements by dividing the current total biomass *X(t)* with the value of the previous measurement *X(t* *−* *1)*. This equation assumes that *µ* is constant for the described time interval.

#### Moving average

A moving average filter was applied to smooth the stepwise integral estimation by calculating the mean of the observations using a fixed window size as stated in the following equation:

3 $$\mu_{{\text{MA }}} = \frac{{\mu_{\left( t \right)} + \cdots + \mu_{{\left( {t + n - 1} \right)}} }}{n},$$

with $\mu_{{{\text{MA}}}}$ as the smoothed value, $\mu$ the growth rate, and the chosen window size *n*.

### Cubic smoothing spline

For the specific growth rate estimation via cubic smoothing spline, the MATLAB function *csaps(x,y,p)* was applied with *x* the total time of the process, the total cell mass *y*, and the chosen value for the fitting parameter *p*. This function is an implementation of the Fortran function SMOOTH [18]. The fitting parameter *p* determines the relative weight to either smooth or perfectly match the data. Here, the least-squares solution (*p* = 0) is a straight line fit, while *p* = 1 is the natural cubic spline interpolation matching each data point. To find the optimal fit, the *p* value was screened with a resolution of 0.1 and applied to the data. By choosing an appropriate value for *p,* the current growth rate can be determined by computing the functions respective time derivative (Eq. 4):

4 $$\frac{{{\text{d}}\left( {x V} \right)}}{{{\text{d}}t}} = \mu x V,$$

with *x* representing the biomass concentration and *V* the volume. The MATLAB script to apply the described cubic smoothing spline function to real data can be found in the Online Resource 2.

### Specific substrate uptake rate

For the calculation of the specific substrate uptake rate in g/g/h (*qS*), different approaches were considered and compared with regard to the respective accuracy. For the following equations, *uf* represents the feed flowrate, *Sf* the substrate feed concentration, *S* the substrate concentration, *V* the volume, and *x* the biomass concentration. The change in substrate over time is determined by the amount of consumed and added substrate in the reactor (Eq. 5), accordingly:

5 $$\frac{{{\text{d}}\left( {S V} \right)}}{{{\text{d}}t}} = qS x V + uf Sf.$$

#### Option 1

For the first approach, the total substrate consumption (i.e., accumulation minus input) was calculated and set into a relationship to the *qS* (Eq. 6). Accordingly, rearranging and integrating Eq. (5) resulted in:

6 $$\frac{{{\text{d}}(S V - S_{0} V_{0} - \smallint uf Sf {\text{d}}t)}}{{{\text{d}}t}}\frac{1}{x V} = qS.$$

A cubic smoothing spline fit was performed on the total consumption ($S V - S_{0} V_{0} - \smallint uf Sf{\text{ d}}t$) and on the biomass term $\left( {x V} \right)$.

#### Option 2

For the second approach, the total amount of substrate in the supernatant was taken into consideration for the spline function and set into relation with the *qS* (Eq. 7). The cubic smoothing spline fit was performed on the substrate term $\left( {S V} \right)$ and on the biomass term $\left( {x V} \right)$:

7 $$\left( {\frac{{{\text{d}}\left( {S V} \right)}}{{{\text{d}}t}} - uf Sf} \right) \frac{1}{x V} = qS.$$

#### Option 3

The last approach is similar to the second one, but only takes the substrate concentration in the supernatant into account. Accordingly, it follows from Eq. (5):

8 $$\begin{gathered} \frac{{{\text{d}}\left( {S V} \right)}}{{{\text{d}}t}} = V\frac{{{\text{d}}S}}{{{\text{d}}t}} + S\frac{{{\text{d}}V}}{{{\text{d}}t}} = qS x V + uf S, \hfill \\ {\text{with}} \frac{{{\text{d}}V}}{{{\text{d}}t}} = uf, \hfill \\ \end{gathered}$$

9 $$\begin{gathered} V\frac{{{\text{d}}S}}{{{\text{d}}t}} - uf Sf + uf S = qS x V, \hfill \\ {\text{with }} D = \frac{uf}{V}, \hfill \\ \end{gathered}$$

10 $$\left( {\frac{{{\text{d}}S}}{{{\text{d}}t}} - D\left( {Sf - S} \right)} \right)\frac{1}{x V} = qS.$$

For this, an additional variable must be introduced, the dilution rate *D*, which is defined as the ratio of *uf* to *V* (Eq. 10). The cubic smoothing spline fit was performed on the substrate concentration term $\left( S \right)$ and on the biomass term $\left( {x V} \right)$.

### RMSE and MAPE calculation

The root-mean-square error (RMSE) was calculated according to Eq. (11) and the mean absolute percentage error (MAPE) according to Eq. (12), where $\hat{y}$ describes the actual value, $y$ the desired target value and *n* the number of samples:

11 $${\text{RMSE}} = \sqrt {\frac{{\mathop \sum \nolimits_{t0}^{i} (\hat{y}\left( t \right) - y\left( t \right))^{2} }}{n}} ,$$

12 $${\text{MAPE}} = \frac{{\sum \frac{{\left| {y\left( t \right) - \widehat{y\left( t \right)}} \right|}}{y\left( t \right)}}}{n} \times 100.$$

## Results

### Bioprocess simulation

**Fig. 1:**
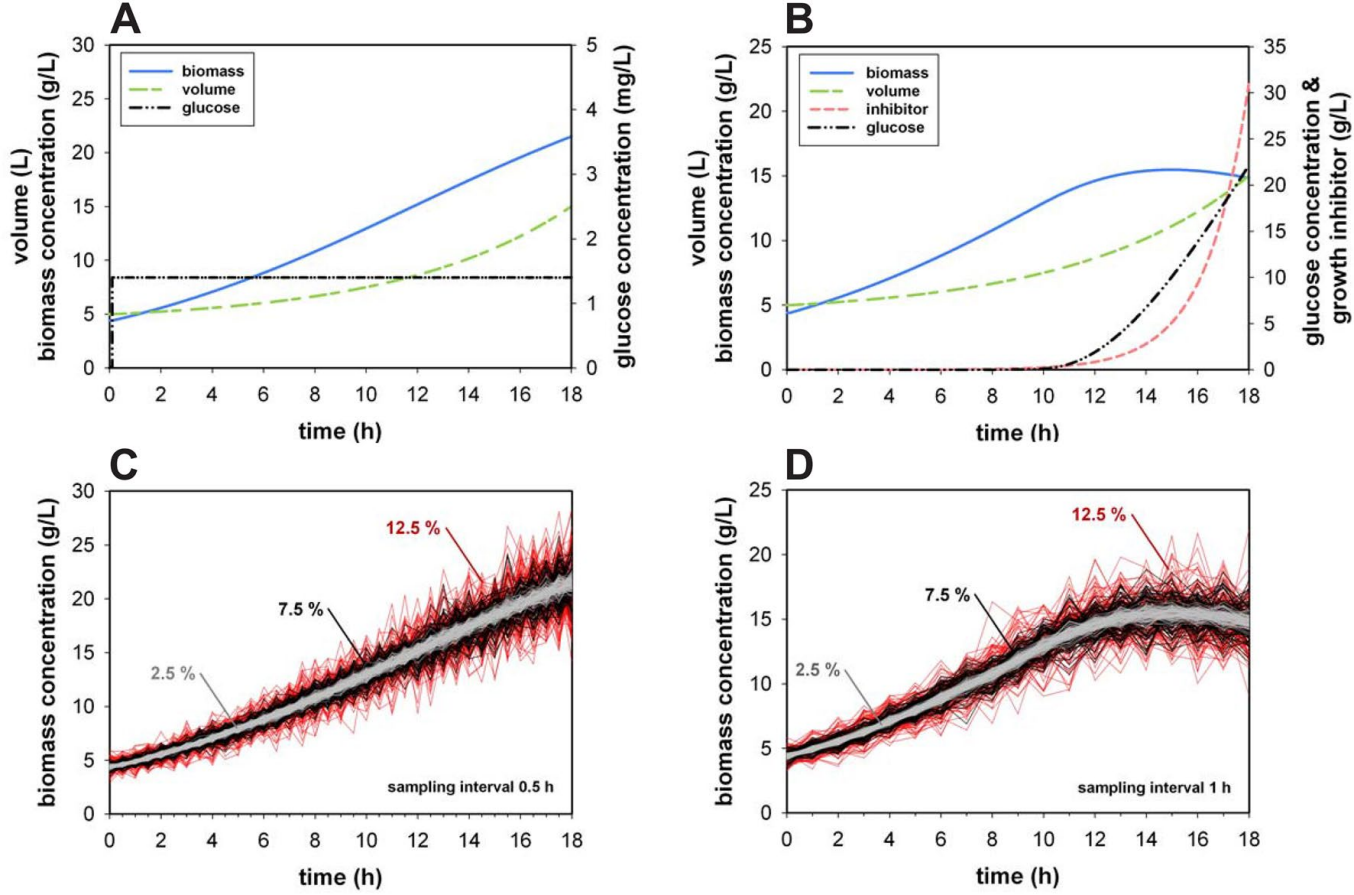
Simulated **a** Monod and **b** non-competitive model process parameters and biomass concentration variation due to random sampling error at 12.5%, 7.5% and 2.5% CV for the Monod model (**c**) with a sampling interval of 0.5 h and the non-competitive model (**d**) with a sampling interval of 1 h are presented. For **c**, **d** the number of simulated fed-batch processes *n* = 100

The two different bioprocess setups are displayed in Fig. 1. Simulation 1 describes a bioprocess were the cells are not induced or do not exhibit any growth inhibition (Fig. 1a). The second simulation describes a typical biomass trend of an induced microbial process (Fig. 1b). Due to this setup, we obtained completely different trends for the biomass as well as for the substrate concentrations. This allows to test if the distinct curvature of those trends leads to any unwanted effects when the different methods calculating the growth rate are applied.

When a process is performed with exactly the same process parameters for an infinite number of runs and with the exact same time interval at which samples are drawn, still random errors are likely to occur. Due to the analytical method precision, which depends on the utilized device different amounts of CV can be expected. The CV of biomass determination, for instance, is obviously depending on the used method. Gravimetric dried biomass determination for *E. coli* is expected to be quite accurate, whereas the measurement of the viable cell count via a microscope using a hemocytometer can be rather imprecise [1, 2]. The generated variations between 2.5 and 12.5% already represent very precise cell measurements. For instance, at 7.5% CV, the biomass at 20 g/L varies with ± 1.5 g/L, which is an absolutely realistic value (see Fig. 1c, d).

### Rate estimations via stepwise integral estimation and elucidation of sampling interval impact

**Fig. 2:**
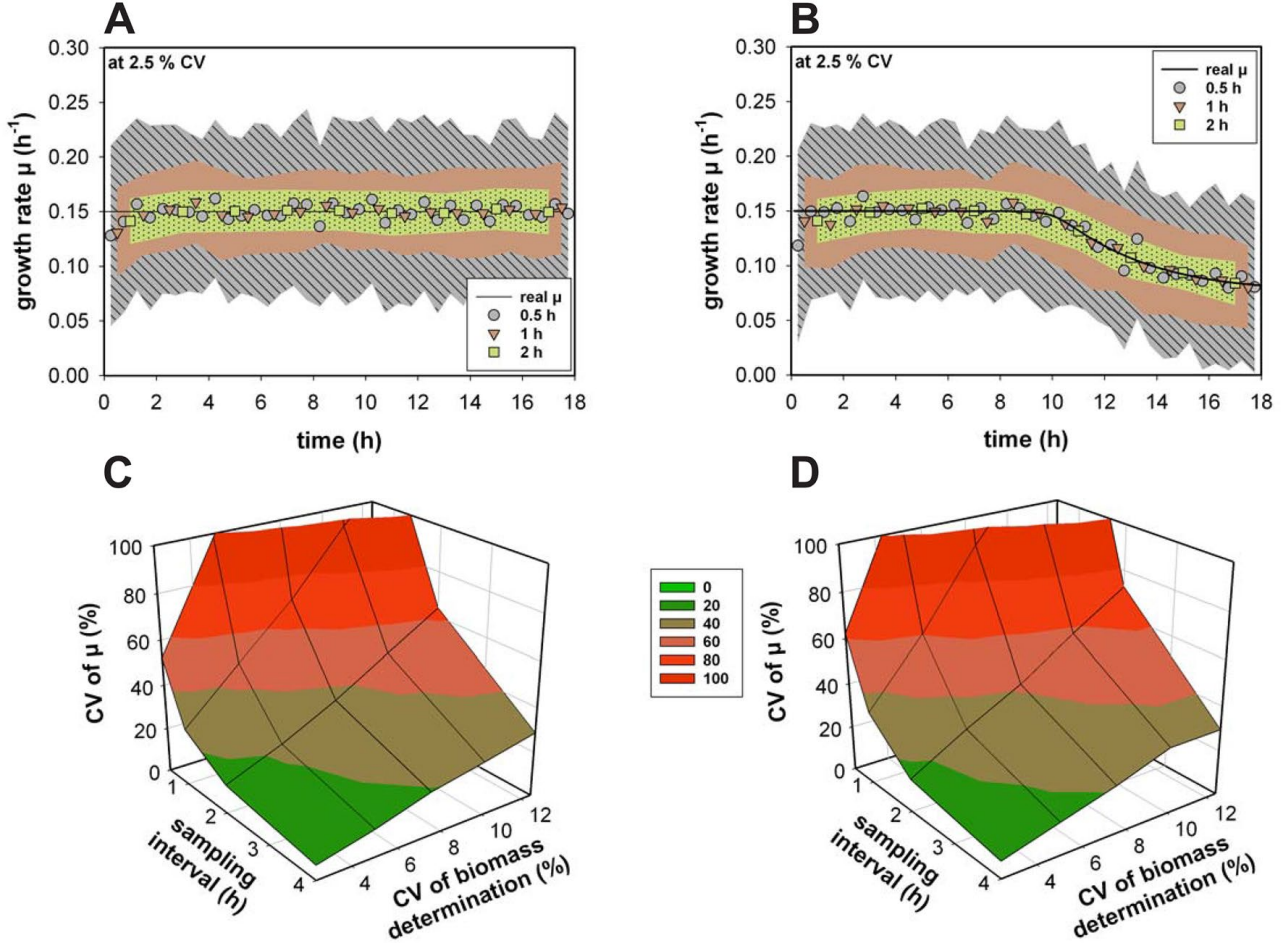
**a**, **b** The estimated growth rates at different sampling intervals and their respective standard deviations (depicted by the area) at a biomass determination precision of 2.5% coefficient of variation (CV). **c**, **d** The resulting CV of the growth rate *µ* as a function of the sampling interval and at different biomass determination precisions for Monod model (**a**,** c**) and the non-competitive model (**b**,** d**) The number of simulated processes *n* = 100. Data above 100% are not depicted

In the first step, the growth rates for the 100 simulated fed-batch experiments were calculated and the accuracy and precision of the growth rate estimations were determined. For each rate *µ(i)* at time point *t(i)*, the average and the standard deviation were calculated (n = 100). On average, the stepwise integral estimation is able to determine the rate quite precisely, independently if the growth rate is constant (Fig. 2a) or not (Fig. 2b). However, it is attended by low accuracy and further depends on the sampling interval and biomass accuracy. At an interval of 0.5 h, for instance, the minimal CV is already around 50% (Fig. 2c, d). Additionally, at a low biomass determination accuracy, the CV even increases fivefold. If the growth rate is following a dynamic trend, the maximum CV at the highest sampling frequency is almost 400%. For both bioprocesses, the CV for almost half of the dataset was higher than 50%.

This behavior of the stepwise integration has huge implications on the evaluation of the current growth rates. For instance, if the growth rate would be rapidly changed back and forth due to a modification in the experimental condition, the stepwise integration approach would not be able to recognize this and the information would remain hidden because of the weak performance.

### Rate estimation via cubic smoothing spline

**Fig. 3:**
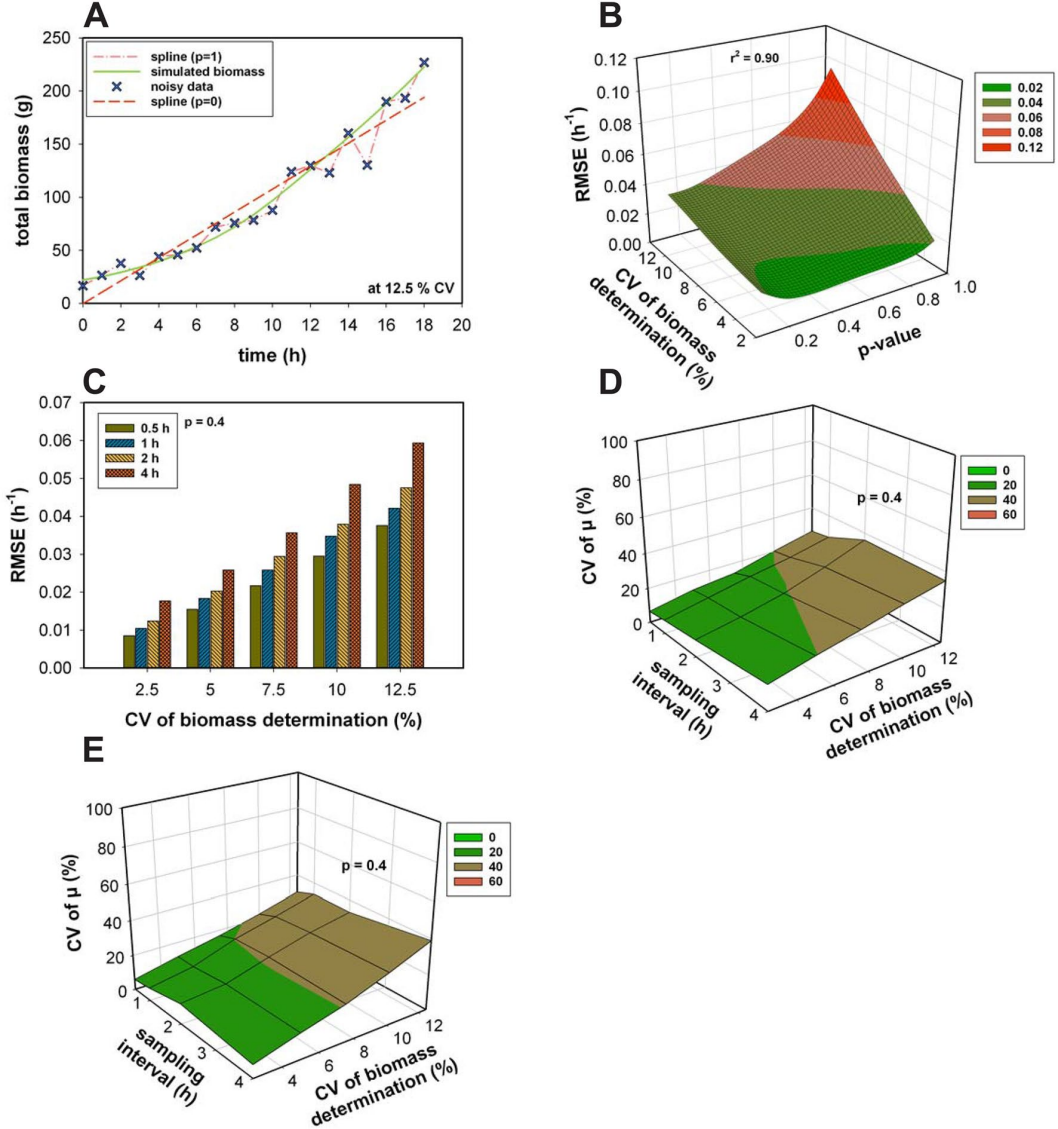
**a** Spline fittings with *p* 0 and 1 of noisy biomass data (12.5% CV of biomass determination). **b** RMSE as a function of the sampling interval, the CV of biomass determination and the fitting parameter *p* of the spline function. **c** RMSE at a *p* of 0.4 at different sampling intervals. The coefficient of variation (CV) of the growth rate for the Monod model (**d**) and the non-competitive model (**e**) as a function of the sampling interval and CV of biomass determination for a fitting parameter *p* of 0.4. For **b**–**e** the number of simulated processes *n* = 100

The cubic smoothing spline function was applied to the whole data for each run. The performance of the smoothing spline curve is displayed in Fig. 3. Additionally for the smoothing spline, also the perfect value for a general purpose of *p* was screened. A fitting parameter *p* of 1 led to a very low error but also to a generalization of the data and a *p* of 0 to an increasingly high error due to the simple straight line fit (Fig. 3a). Therefore, both were not displayed in Fig. 3b. To obtain the optimal *p*, the RMSE (Eq. 11) of the rates for 100 simulated fed-batch experiments at different sampling frequencies and CV for biomass determination was calculated (Fig. 3b) and described as a function of *p*, added noise, and sampling frequencies. The RMSEs of all the sampling intervals resulted in a similar shape. The surface exhibited a minimum at a *p* around 0.4 for all noise and sampling frequency combinations except for noise levels > 10% and the lowest sampling frequency of 4 h where a slightly lower *p* of 0.2 would be more preferable (see also Fig. 3c). Consequently, a fitting parameter of 0.4 was chosen for all further processes. At this magnitude, also the overall error at high sampling intervals and large measurement errors is reasonable low. Once the fit is applied sufficiently, the time derivative of this function represents the current growth rate. A very precise and accurate fit can be generated, which is sampling interval independent using the applied smoothing spline function. Even if the rate estimations became slightly inaccurate at the beginning and at the end of the processes, still the precision for the rate estimations via spline is high. No differences between the estimation of a constant and a decreasing growth rate were evident. Also, if large noise was present, the spline was still able to estimate the rates correct and precise (Fig. 3d, e). With a biomass measurement error of 12.5%, the calculated CV ranged around 50% (*n* = 100).

### Methodical comparison: stepwise integral estimation and cubic smoothing spline

The combination of stepwise integration and a moving average is a widely used approach for gathering smoothed rates. In the following, we elucidate the differences of using this combined method with the cubic smoothing spline.

The rate estimations described via the cubic smoothing spline outperformed the stepwise integral estimation. While the spline is considering the whole data, the stepwise integral estimation only takes two consecutive time points into account. Hence, smoothing splines can better deal with the error in the data compared to stepwise integral estimations. Regarding stepwise integral estimation, the error in the data is further propagated into the rate calculation. The spline fit already smooths the data before it gets even further processed. Considering this fact, it is obvious that spline functions are more accurate and precise.

**Fig. 4:**
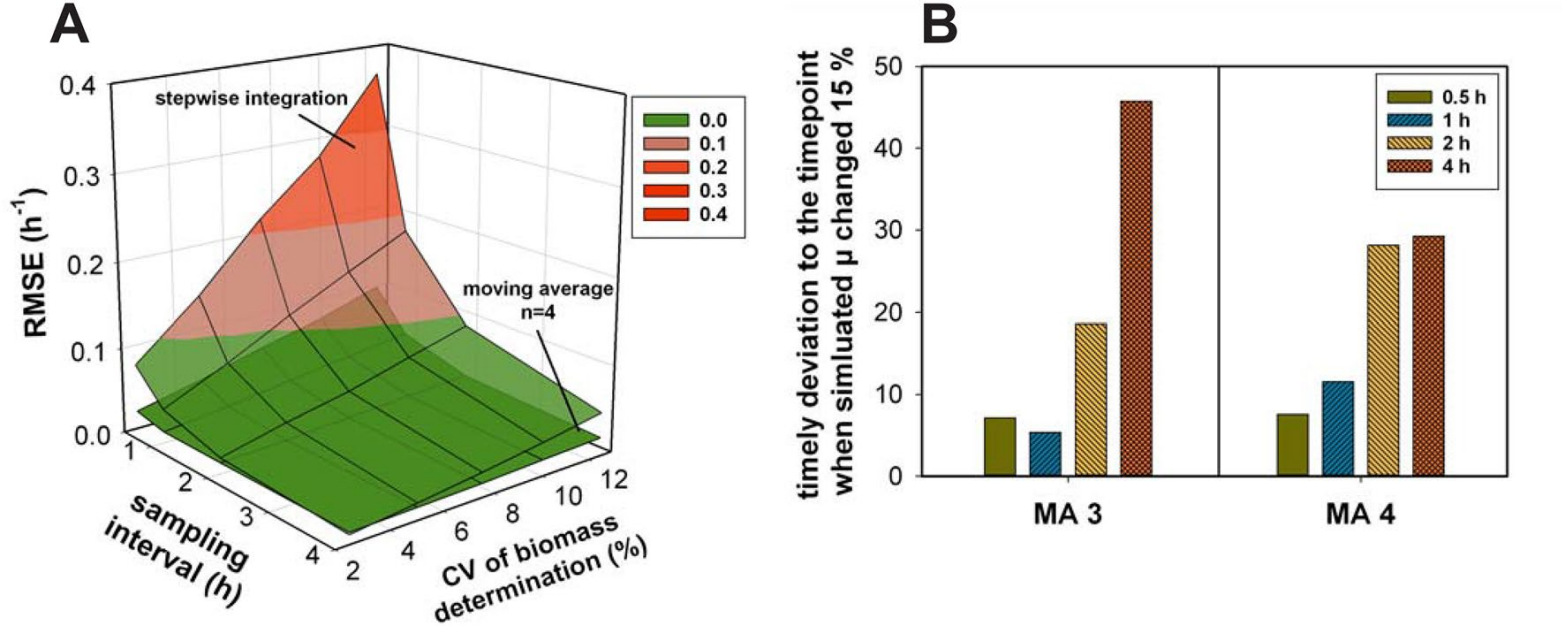
Comparing the RMSE values of the stepwise integral estimations (**a**) and stepwise integral estimations using a moving average (*n* = 4) as a function of the sampling interval and CV of biomass determination. **b** The timely deviation (%) from the time point when the simulated *µ* changed 15% (non-competitive model) derived from utilizing moving average with a window size of 3 and 4. The number of simulated processes *n* = 100

A very common approach to further process the rates derived from stepwise integral estimations is to apply a moving average filter to smooth the data. For this study, we have chosen an averaging window size of 3 and 4. As expected the larger is the window size, the smaller the variations. Even with a window size of 3, the RMSE was reduced to an acceptable level. At a window size of 4, the error in the rate estimations in some cases was even better than the ones calculated with the cubic smoothing spline (Fig. 4).

However, due to the moving average, the rate change will seem to occur at different time points than it is the case. This is, in particular, a problem for non-constant rates (Fig. 4b). This effect will get even stronger at lower sampling frequencies. Further, averaging rates over several time points reduces the ability to describe the dynamics in the system, whereas exactly this should be described by the rates. The more likely process changes occur and the larger the averaging window is, the more likely they are overseen. Hence, the increased precision is traded for a reduced rates description.

The user also has to face the so-called endpoint problem. Due to the application of the moving average, the end of the process is not determined. Depending on the window size, the timeline of the rates will be inevitable shorter. Consequently, the utilization of moving average will reduce variation in the prediction, but will also lead to a reduced descriptiveness of the process and to misleading assumptions.

### Specific substrate uptake rate estimations via the cubic smoothing spline

**Fig. 5:**
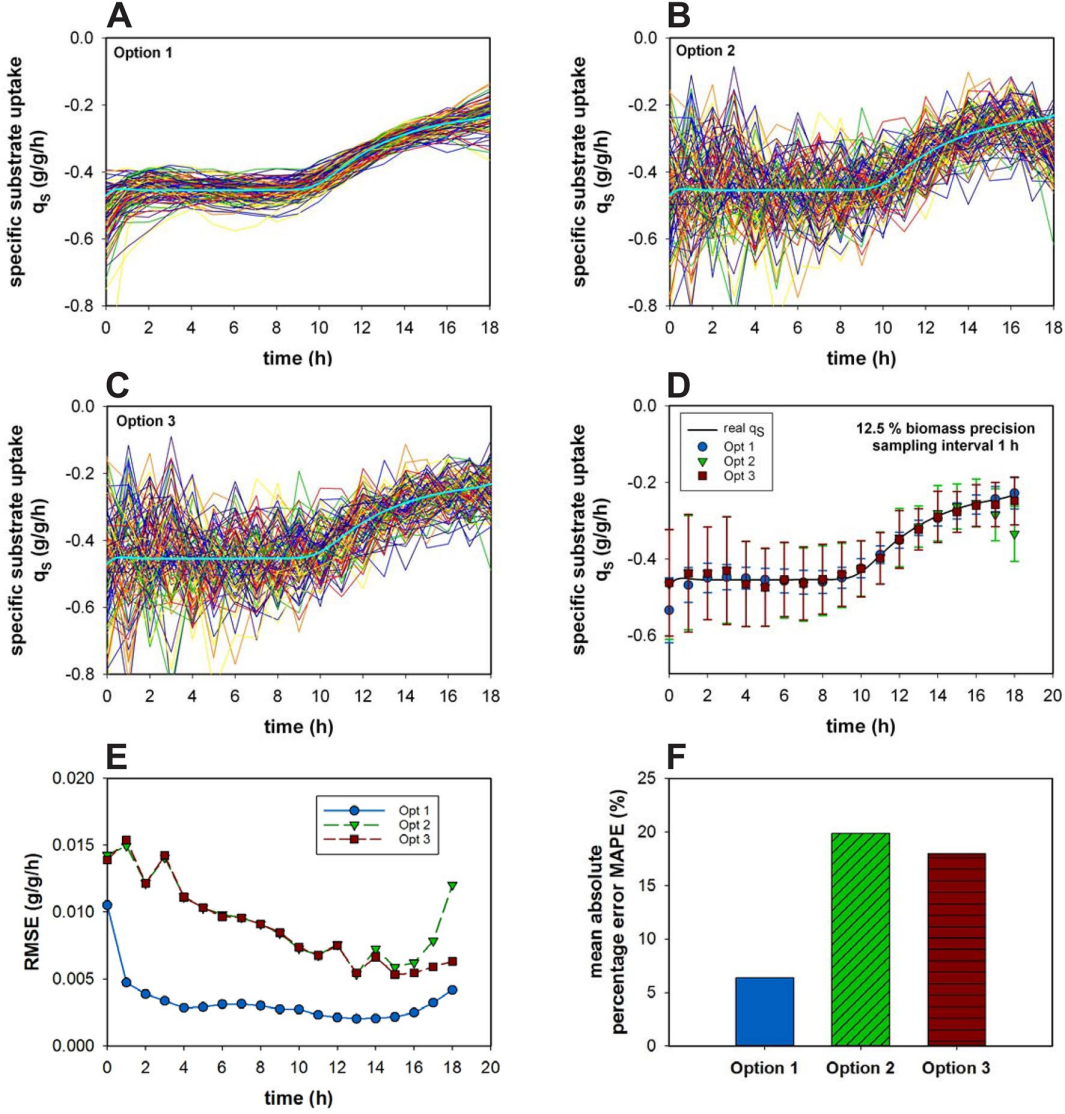
Specific substrate uptake rate estimation via option 1 (**a**) 2 (**b**) and 3 (**c**) over the time course of a fed-batch (*n* = 100) for a sampling interval of 1 h and precision of 12.5% CV for the biomass determination are presented. The averaged values and their respective standard deviations of the three different options over the time course of the process (**d**), the resulting RMSE values for each option and sampling point (**e**), and MAPE for all three options (**f**) are displayed. The number of simulated processes *n* = 100

Other important process characteristics are substrate uptake rates. In this specific case, the amount of fed substrate must be incorporated into the calculation and with it any possible variations and errors, which might come along. Since we already verified the superiority of a cubic smoothing spline we only focused on the performance of this approach. A simulation of 100 fed-batch processes using the non-competitive model was performed in which a feed variation of 1% occurs. The sampling interval was chosen to be 1 h and the worst case of 12.5% CV for the biomass determination was used and the fitting parameter *p* was set to 0.4. There are three possible options for the estimation of a feed-dependent rate. Either the total amount of consumed substrate (Option 1), the total amount of substrate in the supernatant (Option 2) or the substrate concentration in the supernatant (Option 3) can be taken into consideration for the cubic smoothing spline fitting (Fig. 5a–c).

All three options can in average accurately describe the specific substrate uptake rate (Fig. 5d). However, the incorporation of the feed into the calculation beforehand increased the precision to a great extent (Option 1) and also the feeding noise can be almost completely erased. Interestingly, between option 2 and 3, respectively, using the total amount of substrate or the substrate concentration, no significant difference was observed (see Fig. 5e). Only at the end of the fed-batch process, option 2 underestimates the specific substrate uptake rate. However, already 1% variation in the feeding system can have a substantial impact. As a consequence of using the wrong approach, the error will increase almost fourfold (Fig. 5f) from around 5% up to 20% MAPE (Eq. 12). If the feed is not incorporated into the calculation beforehand, such as it is the case in Option 2 and 3, the feeding error propagates further into the rate estimation.

## Discussion

### Stepwise integral estimation issues

The key to process development and process modeling is to estimate rates accurately and precisely. In average (*n* = 100), the stepwise integral approach calculated an accurate rate value. This was expected considering that a large number of repetitive experiments should always meet in average the desired target value. But, we demonstrated that the stepwise integral estimation will end up in large variations. It is not surprising that the inaccuracy rises with an increased sampling frequency [24], but such an increasing variation at higher sampling frequencies was on first sight rather unexpected. Due to the magnitude of the sampling errors, the slope of the linear function will either be more positive or negative, in comparison to the real value. Every new sampling point will add its failure to it and, consequently, the deviation will increase over the time course of the cultivation. Therefore, with an increased sampling frequency, the rate estimation error increases although the measurement error remains constant. Since this behavior is counterintuitive, it is most likely overseen. This is a major disadvantage since for accurate process characterization and to gather process know-how a large dataset, thus a high sampling frequency, is a necessity. The application of the moving average would be a simple tool to reduce such variances but the user will eventually end up in less accurate values. Therefore, rates calculated by stepwise integral estimation should be handled carefully for modeling purposes.

### Application of cubic spline and specific substrate rate estimation

In this study, we focused on the cubic smoothing spline function as an alternative to rate estimations via stepwise integral estimation. With a reduced precision of the analytical determination, also the variation in the estimation increased but not to the same extent as when the stepwise integral estimation was applied. In the best case, at a high sampling frequency and biomass determination inaccuracy, the CV was around a factor of 4 lower. Moreover, the cubic smoothing spline was not affected by the sampling frequency. In real bioprocesses, a good trade-off between sampling frequency, process dynamics and the analytical error should be considered. For high analytical errors and slow process dynamic changes, a high sampling interval does not increase precision and accuracy.

Additionally, we elucidated three different approaches for estimating substrate uptake rates via the established spline fit. If the substrate feed is not incorporated beforehand a cubic spline is performed, feed variations can have a substantial impact on the propagated error. Hence, it is important to first calculate the total amount of consumed substrate before the rates are estimated.

The only “drawback” using the cubic smoothing spline function is that one degree of freedom is present, the fitting parameter *p*. Therefore, before processing the optimal *p* must be reconsidered with respect to the given magnitude of the *x* ordinate. Another powerful alternative to spline functions can be found in Gaussian distributions. It was shown that for processes with high sampling numbers (100–1000), the Gaussian distribution outperforms the spline function while for samplings below 100, it is vice-versa [21]. Typically, mammalian cell culture processes lead to only 10–20 observations. Likewise, also microbial fermentations do not comprise such a high sampling frequency, also resulting in only 15–25 observations per process. These considerations and the remarkably easy use of this method due to no data pre- or post-processing are clearly stating the advantage of the smoothing spline compared with other methods.

## Conclusion

In this study, the specific growth rate and the specific substrate uptake rate were chosen as representative examples. It was shown that cubic spline estimations are a simple but powerful tool to determine rates, compared to the most commonly used standard procedure the stepwise integral estimation. The presented method:

- is easy to apply and to implement for off-line analytical purposes,
- is to a major extent sample interval independent,
- can cope with large analytical variances,
- allows the user to assess a rate value at every time point.

In addition, we showed that a small error in the feeding system can lead to huge impacts in the estimation of specific substrate uptake rates. Hereby, it is important to take the feeding into account before the actual spline fit takes part.

For this level of complexity, the spline is sufficiently enough and more complex algorithms such as the Gaussian distribution or functions with more degrees of freedom (e.g., Kalman filters) are not necessary. It is easy to implement into existing codes and can add a reasonable value to process development and process comparability.

## Electronic supplementary material

Below is the link to the electronic supplementary material.

Supplementary file1 (PDF 88 kb)

Supplementary file2 (PDF 26 kb)
