# Supplementary material for: The shortcomings of accurate rate estimations in cultivation processes and a solution for precise and robust process modeling
Source: Bioprocess Biosyst Eng. 2019 Sep 20;43(2):169–78. doi: 10.1007/s00449-019-02214-6 (PMC6960212; doi:10.1007/s00449-019-02214-6)
Supplement: Supplementary file 1 — Supplementary file1 (PDF 88 kb) [file 449_2019_2214_MOESM1_ESM.pdf]

Article title: The shortcomings of accurate rate estimations in cultivation processes and a solution for precise and robust process modeling

Journal name: Bioprocess and Biosystems Engineering

Author names: *Bayer B.*[1*], *Sissolak B.*[2*], *Duerkop M.*[1], *von Stosch M.*[3], *Striedner G.*[1]

Affiliations:

[1] Department of Biotechnology, University of Natural Resources and Life Sciences Vienna, Austria

[2] Bilfinger Industrietechnik Salzburg GmbH Salzburg, Austria

[3] School of Chemical Engineering and Advanced Materials, Newcastle University, Newcastle upon Tyne, United Kingdom

* These authors contributed equally.

Corresponding authors:

benjamin.bayer@boku.ac.at, +43 1 47654-79169

bernhard.sissolak@boku.ac.at, +43 1 47654-79171

Online Resources

Bioprocess Simulation

We simulated the fed-batch phase of an *E. coli* fermentation for two different process setups using MATLAB (2016b, MathWorks, Massachusetts, USA). Each simulation started with the indicated parameter values as stated in Table 1. 100 individual fed-batches for each setting were simulated. These processes were simulated with varying sampling intervals of 0.5, 1, 2 and 4 h, respectively and a negligibly small sampling volume. Also, the biomass *X* was calculated as stated in Eq. 1 via the growth rate μ and time *t*.

$$X(t) = X(t_0)\, e^{\mu\,(t-t_0)} \quad (1)$$

Further, the exponential feeding strategy was established as indicated in Eq. 2.

$$uf = \frac{1}{Sf}\, X(t_0)\, Y_{xs}\, \mu\, e^{\mu\,(t-t_0)} \quad (2)$$

With the feed flow rate *uf*, the feed glucose concentration *Sf*, the total biomass at the feed start $X(t_o)$, the biomass per glucose yield Y_{xs} and the set growth rate μ.

Table 1. Cultivation settings for the simulated fed-batch processes.

Parameter	Value
starting biomass concentration	4.4 g/L
vessel volume	5 L
feeding strategy	exponential
growth rate	0.15 h^{-1}
feed duration	18 h
feed glucose concentration	100 g/L
inhibitor concentration	80 g/L
yield biomass/glucose	0.33 g/g
sampling interval	0.5, 1, 2, 4 h

Monod model

The first *in-silico* process setup was based on a glucose limitation and therefore the apparent growth rate was adjusted by the feeding rate only, following the order of Monod (Eq. 3) as shown in Figure 1A.

$$\mu = \frac{\mu_{\max}\ S}{K_S + S} \quad (3)$$

With the specific growth rate μ, the assumed maximum growth rate μ_{max} of 0.9 h^{-1}, the limiting glucose/substrate concentration S, and the substrates affinity constant K_S with an assumed value of 0.007 (Senn, Lendenmann, Snozzi, Hamer, & Egli, 1994).

Non-competitive model

For the second process setup the exponential addition of an inhibitor, mimicking the product formation, was taken into account (Eq. 4). The inhibitor concentration was selected in a way that adequately mimics a decreased growth rate as presented in Figure 1B. Hence, the simulation was performed in such a way that the inhibitor was included in the feed.

$$\mu = \frac{\frac{\mu_{\max} S}{K_S + S}}{1 + \frac{I}{K_i}} \qquad (4)$$

Using the non-competitive model the Monod equation with the same assumed values is extended with an additional term to describe the growth inhibition, containing the inhibitor concentration *I* and its affinity constant K_i with an assumed value of 1.7.
