# Supplementary material for: The shortcomings of accurate rate estimations in cultivation processes and a solution for precise and robust process modeling
Source: Bioprocess Biosyst Eng. 2019 Sep 20;43(2):169–78. doi: 10.1007/s00449-019-02214-6 (PMC6960212; doi:10.1007/s00449-019-02214-6)
Supplement: Supplementary file 2 — Supplementary file2 (PDF 26 kb) [file 449_2019_2214_MOESM2_ESM.pdf]

Article title: The shortcomings of accurate rate estimations in cultivation processes and a solution for precise and robust process modeling

Journal name: Bioprocess and Biosystems Engineering

Author names: *Bayer B.*[1*], *Sissolak B.*[2*], *Duerkop M.*[1], *von Stosch M.*[3], *Striedner G.*[1]

Affiliations:

[1] Department of Biotechnology, University of Natural Resources and Life Sciences
Vienna, Austria

[2] Bilfinger Industrietechnik Salzburg GmbH
Salzburg, Austria

[3] School of Chemical Engineering and Advanced Materials, Newcastle University,
Newcastle upon Tyne, United Kingdom

* These authors contributed equally.

Corresponding authors:

benjamin.bayer@boku.ac.at, +43 1 47654-79169

bernhard.sissolak@boku.ac.at, +43 1 47654-79171

MATLAB script for growth rate estimation via cubic smoothing spline

Depending on the dataset the optimal *p* can differ. We observed a time dependency with respect to *p* using minutes instead of hours for microbial or hours instead of days for mammalian processes, which can lead to different cubic spline results. Thus, to expect optimal fitting results for a fixed *p*, the time axis should be in a similar range. In this case, the optimal *p* of 0.4 is valid for processes in the double-digit range (e.g.: 20 hours or days). Moreover, we performed *csaps* for real microbial as well as cell culture processes and always established a good fitting performance with a *p* of 0.4.

For this section user input is needed - set the value for the fitting parameter p and import your Excel file including your process data for the growth rate calculation

```
clear ; clc ; close all

% create an Excel file, it only has to include the columns for the
 following process parameters in the indicated order to work (exclude
 any headers)
% column A = absolute time of the process
% column B = viable cell concentration/biomass
% column C = vessel volume (in the same unit as the concentration in
 Column B)

% if your file is complete, choose your wanted value for the fitting
 parameter p
fitP = 1 ;

% import your Excel file by assigning it to the variable 'importData'
importData = importdata('testfile.xlsx') ;

% the output of this script will be a newly generated Excel file
% It contains the sampling points with the respective viable cell
 concentration/biomass, total viable cells/biomass and the growth
 rate, all calculated via the cubic smoothing spline function
% the used value for the fitting parameter will also be saved in this
 Excel file
% the file will be created into the same folder as this Matlab script
 is located

% run the code (F5)
```

no further user input is needed for this section - rate calculation and export to Excel file

```
% assigns the variables to the column number in your Excel file
Time_Total = importData(:,1) ;
VCC = importData(:,2) ;
Volume = importData(:,3) ;

% function generation and value calculation
fnX = csaps(Time_Total,VCC.*Volume,fitP);
GrowthRate_Spline_Samplings = fnval(fnder(fnX,1),Time_Total)./
fnval(fnX,Time_Total);

% plots the measured and calculated cell concentration
```

```
figure(1)
plot(Time_Total, VCC.*Volume, 'kd', 'MarkerSize',
 10,'markerfacecolor','k')
title('Measured versus calculated Values')
xlabel('Total Time')
ylabel('Total Viable Cells')
hold on
fnplt(fnX)
legend('Measurement', 'Calculated Trend', 'Location', 'northwest')
hold off

% plots the calculated growth rate via smoothing Spline with the
 chosen fitting parameter p
figure(2)
hold on
plot(Time_Total, GrowthRate_Spline_Samplings)
title('Calculated Growth Rate via Smoothing Spline')
xlabel('Total Time')
ylabel('Growth Rate')
legend('Spline Estimation') ;
hold off

% creates variables for the Excel export
ExcelSheet = 1 ;
Export_Names = {'Total Time - Sampling Points', 'Calculated Biomass/
Viable Cell Concentration via Smoothing Spline', 'Calculated Total
 Biomass/Viable Cells via Smoothing Spline', 'Calculated Growth Rate
 via Smoothing Spline',  'used Value of the Fitting Parameter'} ;

TVC_Calculated = fnplt(fnX) ; TVC_Calculated = TVC_Calculated' ;
alignment = knnsearch(TVC_Calculated(:,1),Time_Total) ;
VCC_Calculated = TVC_Calculated(alignment,2)./Volume ; TVC_Calculated
 = TVC_Calculated(alignment,2) ;

% creates Excel export file
xlswrite('SmoothingSpline_Calculations.xlsx', Export_Names,
 ExcelSheet, 'A1:E1') ;
xlswrite('SmoothingSpline_Calculations.xlsx', Time_Total,
 ExcelSheet, 'A2') ;
xlswrite('SmoothingSpline_Calculations.xlsx', VCC_Calculated,
 ExcelSheet, 'B2') ;
xlswrite('SmoothingSpline_Calculations.xlsx', TVC_Calculated,
 ExcelSheet, 'C2') ;
xlswrite('SmoothingSpline_Calculations.xlsx',
 GrowthRate_Spline_Samplings, ExcelSheet, 'D2')
xlswrite('SmoothingSpline_Calculations.xlsx', fitP,
 ExcelSheet, 'E2') ; clearvars
```

Published with MATLAB® R2016b
